# Personalized multifactorial risk assessment in neoadjuvant-treated breast carcinoma

**DOI:** 10.1007/s10549-024-07584-4

**Published:** 2024-12-30

**Authors:** K. Korpinen, T. A. Autere, J. Tuominen, E. Löyttyniemi, N. Eigeliene, K. Talvinen, P. Kronqvist

**Affiliations:** 1 Institute of Biomedicine, University of Turku, Kiinamyllynkatu 10/MedD5A, 20500 Turku, Finland; 2 Department of Pathology, Turku University Hospital, Turku, Finland; 3 Department of Biostatistics, University of Turku, Turku, Finland; 4 Department of Oncology, Vaasa Central Hospital, Vaasa, Finland

**Keywords:** Breast cancer, Neoadjuvant, Prognosis, Biomarker, Risk evaluation

## Abstract

**Purpose:**

Due to biological heterogeneity of breast carcinoma, predicting the individual response to neoadjuvant treatment (NAT) is complex. Consequently, there are no comprehensive, generally accepted practices to guide post-treatment follow-up. We present clinical and histopathological criteria to advance the prediction of disease outcome in NA-treated breast cancer.

**Methods:**

A retrospective consecutive cohort of 257 NA-treated Finnish breast cancer patients with up to 13-year follow-up and the corresponding tissue samples of pre- and post-NAT breast and metastatic specimen were evaluated for prognostic impacts. All relevant clinical and biomarker characteristics potentially correlated with tumor response to NAT, course of disease, or outcome of breast cancer were included in the statistical analyses.

**Results:**

The results highlight the intensified characterization of distinguished prognostic factors and previously overlooked histological features, e.g., mitotic and apoptotic activity. Particularly, decreased PR indicated 3.8-fold (CI 1.9–7.4, *p* = 0.0001) mortality risk, and a > 10.5-year shorter survival for the majority, > 75% of patients (Q1). Clinically applicable prognostic factors both preceding and following NAT were identified and compiled into heat maps to quantify mortality and recurrence risks. Combinations of risk factors for aggressive disease were exemplified as an interactive tool (bcnatreccalc.utu.fi) to illustrate the spectrum of disease outcomes.

**Conclusion:**

The results emphasize the value of comprehensive evaluation of conventional patient and biomarker characteristics, especially concerning re-assessment of biomarkers, risk-adapted surveillance, and personalized treatment strategies. Future personalized NA-treatment strategies might benefit from models combining risk-adapted surveillance data and post-NAT re-assessed biomarkers.

**Supplementary Information:**

The online version contains supplementary material available at 10.1007/s10549-024-07584-4.

## Introduction

Neoadjuvant therapy (NAT) is the standard initial treatment of locally advanced breast carcinomas representing 3–30% of all new primary breast cancer diagnosis [1, 2]. In current practice, NAT indications have extended to comprise patients with high-risk early-stage disease, especially Human epidermal growth factor receptor 2 (HER2)-amplified and triple-negative breast carcinomas (TNBCs) [3, 4]. According to literature, NAT is equally efficient to adjuvant treatment [5]. NAT has additional benefits in guiding individualized therapies, particularly providing information on the chemosensitivity of the tumor and eradicating occult lesions, enabling radical surgery and/or breast-conserving techniques, and, consequently, improving postoperative quality of life [6]. Currently, only limited data are available for predicting risk of disease recurrence post-NAT. Consequently, there are no generally accepted post-treatment practices to guide follow-up or modify adjuvant interventions.

Due to clinical and biological heterogeneity of breast carcinoma (BC), patients respond to NAT individually and, occasionally, unpredictably [7]. The fraction of patients achieving pathologic complete response (pCR) varies between 19 and 60%, and 5–20% experience disease progression [8]. pCR is considered to predict favorable course of disease and outcome [9] but, in case of incomplete treatment response, a delay in surgical treatment may adversely affect the patient's survival [10]. In previous literature, the main factors predisposing NA-treated patients for local recurrence have mainly been related to the size and location of the tumor, the principles of evaluating tumor regression, and the adequacy of the surgical margins [5, 11]. However, the available criteria have limited utility for personalized response prediction.

At present, NAT decisions and treatment schema rely on the histopathological classification, especially intrinsic subtypes [4, 12]. To date, there are limited published data on more comprehensive and detailed evaluation of immunohistopathological features in association with NAT, particularly in the post-NAT setting [13, 14]. Especially, there is a lack of research to demonstrate the alterations in biomarker status from pre- to post-NAT settings and their prognostic value [14–16]. Moreover, there is no generally accepted routine for post-NAT retesting of biomarkers [14, 16–18].

This study set out to identify prognostic models for breast cancer both prior to and following NAT. Our results indicate the potential of standardized and intensified evaluation of clinical and biomarker characteristics in predicting disease outcome. To cover the research gap concerning pre- to post-NAT biomarker discordance, we also evaluated and identified systematic alterations in expression status of major prognostic and predictive biomarkers. The results emphasize the value of previously underrated histological features, such as mitotic and apoptotic activity as well as progesterone receptor (PR) expression. To demonstrate the spectrum of different outcomes, the findings were combined into an interactive model composed of cumulative recurrence risks (bcnatreccalc.utu.fi).

## Materials and methods

The study comprises a real-world data of 257 consecutive patients diagnosed with primary invasive BC and treated with NAT at Turku University Hospital Expert Responsibility Area, Finland, during 2010–2020 (Table 1). The treatment indications and protocols followed the standardized Finnish National NA-treatment regimens at the time of diagnosis (Supplementary Table 1), the majority of patients receiving chemotherapy (98.1%), most commonly taxanes alone or combined with anthracycline. In addition, HER2-amplified tumors received HER2-targeted treatment. Breast and axillary surgery, histopathological examination, and adjuvant treatment followed the national guidelines at the time of diagnosis. Complete clinical data were available from patient files and pathology reports. Causes of death were obtained from autopsy reports and Finnish Cancer Registry resulting in a maximum follow-up period of up to 13 years and 4 months (mean 5 years and 8 months).

**Table 1:** Clinico-pathological characteristics at time of diagnosis (*n* = 257)

Patient characteristics	
Mean age at diagnosis (year) (range)	56 (28–84)
Menopausal status (%)	
Premenopausal	35.1
Perimenopausal	2.7
Postmenopausal	62.3
Lactating (%)	3.1
Hormone replacment therapy (%)	14.8
Mean BMI (range)	27.9 (16.9–46.1)
Detected by screening (%)	8.6
Previous malignant disease (%)	3.1

Tumor characteristics	
Mean tumor size (cm) (range)	6.0 (0.6–15.7)
T classification (%)	
T1	4.6
T2	23.3
T3	39.7
T4	32.1
Tx	0.4
N + suspicion in imaging (%)	79.8
Mean diameter of N+ (cm) (range)	2.5 (0.5–6)
Distant metastasis (%)	
M0	87.2
M1	11.7
Mx	1.2
Inflammatory disease (%)	29.0
Histological type (%)	
Infiltrating ductal	77.1
Infiltrating lobular	20.2
Special type	2.7
Her2-positive (%)	39.7
Intrinsic subtype (%)	
Luminal A	5.7
Luminal B (HER2-negative)	40.8
Luminal B (HER2-positive)	29.8
HER2-positive (non-luminal) (%)	9.9
Triple-negative	13.0
Histological grade (%)	
Grade 1	5.0
Grade 2	37.0
Grade 3	56.5
Treatment response (%)	
RCB 0	24.8
RCB I	11.1
RCB II	33.6
RCB III	20.0

Follow-up and survival (%)	
Recurrence	25.6
Breast cancer-specific death	30.4

Archival tissue samples, representing each patient, were collected from the original diagnostic biopsy (pre-NAT specimen) as well as surgical specimen from the tumor bed in the breast and possible axillary metastases (post-NAT specimen) (Supplementary Fig. 1). The specimen was prepared according to the standard histology practice i.e., fixed in buffered formalin (pH 7.0) and embedded in paraffin. Whole tissue sections of all specimen were morphologically re-evaluated (KK, PK) according to WHO [19] and international guidelines [20], as summarized in Table 2. Treatment responses were re-categorized using Residual Cancer Burden (RCB) Calculator (https://www3.mdanderson.org/app/medcalc/index.cfm?pagename=jsconvert3) for pathological complete response (pCR, RCB 0) and partial response (non-pCR, RCB 1–3).

**Table 2:** Summary of evaluated morphological and immunohistochemical features

	Pre-NAT breast biopsy	Post-NAT breast specimen	Post-NAT lymph node specimen
	(*n* = 257)	Cases with residual disease (*n* = 184)	Cases with ypN+ (*n* = 156)
Tumor size (mm) (mean, range)		48 (0.7–200)*	
Size of invasive growth (mm) mean (range)		39 (0.5–200)	
In situ component (%)		39.4	
Micropapillary growth (%)	6.2	16.8	10.9
Mucinous differentiation (%)	1.9	1.6	3.2
Histological grade			
Grade 1	5.0	10.9	
Grade 2	37.6	54.3	
Grade 3	57.4	34.8	
Nuclear grade (%)			
Grade 1	1.1	3.8	
Grade 2	33.3	51.1	
Grade 3	65.5	45.1	
Mitotic count (mean, range)	22 (0–254)	17 (0–146)	21 (0–230)
Apoptosis (%)	66.3	29.9	41.7
Tumor necrosis (%)		17.4	
LVI^a^ (%)		37.8**	
TIL^b^			
Present (%)	54.7	38.0	
Mean (range)	6.8 (1–80)	5.3 (1–80)	
ER-IHC			
> 1% (%)	74.2	79.4	78.2
Median (range)	55.6 (1.1–99.9)	64.4 (1.2–100)	63.6 (1–99.8)
PR-IHC			
> 1% (%)	62.7	59.8	55.1
Median (range)	36.8 (1.3–98.7)	25.4 (1.1–98.8)	19.6 (1.2–98.4)
Ki-67-IHC (%) median (range)	39.3 (2.5–94.2)	14.9 (0–72.6)	17.4 (0–84.6)
Her2-amplified (%)	39.7	15.0	18.7
Intrinsic classification			
Luminal A	5.8	49.1	45.8
Luminal B (HER2 −)	41.1	19.7	18.1
Luminal B (HER2 +)	30.0	11.0	14.8
HER2-amplified (non-luminal) (%)	10.0	4.0	3.9
Triple-negative	13.1	16.2	17.4
Lymph node metastasis (%)			60.5**
Largest diameter (mm) mean (range)			11.3 (0.2–70)
Lymph node ratio (%) mean (range)***			49.7 (3.3–100)
Isolated tumor cells only (%)			3.1**
Extracapsular invasion (%)			35.5**
Lymph node regression (%)****			52.3

*Invasive and/or in situ

**All cases, including pCR

***Fraction of metastatic versus all excised axillary lymph nodes

****Regression defined as presence of macrophages, fibrosis and/or their combination

^a^LVI, Lymphovascular invasion

^b^TIL, Tumor-infiltrating lymphocytes

Pre-NAT biopsies were evaluated as whole sections. Post-NAT specimens from the residual breast tumor and metastasis were collected in tissue microarrays (TMAs), first, by identifying the representative cancer cell areas in H&E stained sections and, then, punching 1.5 mm cores from the corresponding areas in tissue blocks. Immunohistochemical stainings (IHC) for ER, PR, Ki-67, and Her2 were performed according to standard pathology practice using Benchmark ULTRA (Roche Diagnostics Medical Systems, Tucson, AZ, USA) and signals detection using ultraView or OptiView DAB Detection Kit (Roche Diagnostics Medical Systems). HER2 amplification was detected according to ASCO/CAP guidelines using Her2-IHC (intensity score 2+ or 3+) to select cases for verification by HER2/Chr17 double in situ hybridization (ISH) (Ventana HER2 Dual ISH, Roche Diagnostics Medical Systems). ER and PR-IHC (cutpoint at 1% of cancer cells), Ki-67 IHC (cutpoint at 14% of cancer cells), and HER2 amplification status were applied as surrogate indicators for intrinsic classification [19]. Quantification of IHC was performed on digitalized slides (PANNORAMIC 1000, 3DHistech, Budapest, Hungary) using QuPath software [21]. In addition to the established histopathological parameters of invasive BC, we registered the occurrence of mitotic and apoptotic figures, tumor necrosis, as well as specific morphological features, such as micropapillary growth pattern and mucinous differentiation both in biopsy specimen and in the residual breast tumors and lymph node metastases. In addition, in all excised axillary lymph nodes, we evaluated the number and fraction of metastatic lymph nodes (lymph node ratio, LN ratio) [22] and the presence of extracapsular invasion (ECI), as well as features of tumor regression, such as fibrosis and macrophages [23].

### Statistical analysis

Patient characteristics were summarized using descriptive statistics analyzed as continuous variables and classified according to internationally accepted criteria [19]. Patients’ age at diagnosis was statistically divided at mean value (56 years). Patients with M1 disease (*n* = 30) were excluded from prognostic analyses. The studied morphological and immunohistochemical characteristics were analyzed both as continuous variables and allocated into subgroups exhibiting low versus high expression level. The cut-points were optimized based on morphological expression patterns and levels, statistical analyses involving the mean, median, and quartile values of each parameter and, finally, on univariate analyses identifying the most significant survival difference between cancer-specific survival versus mortality in our material. Fisher’s exact tests were applied to detect differences between the studied clinical parameters and tumor characteristics for disease-free (DFS) and breast cancer-specific survival (BCSS). The results of the models were expressed as hazard ratios (HR) of disease recurrence and mortality combined with relative 95% confidence intervals (CI). After the initial selection, the possible variables for multivariable Cox regression analysis were explored using survival trees with ‘rpart’ [24] package for R [25]. Then, a stepwise model selection was carried out, using the ‘survival’ package [26] for testing the Cox models. The least significant variables were removed step-by step, and the AIC values were compared between the models. The final variable selection was achieved by removing variables until no improvement was observed. Possible pairwise interactions between the remaining variables were then tested based on the initial survival trees, with no significant interaction terms remaining the final models. Heatmaps for predicted risk ratios were created using ‘ggplot2’ [27]. Log-rank test and Kaplan–Meier survival curves were used to compare and visualize the disease outcomes based on DFS and BCSS. Initial analyses were performed using JMP 17.0 (JMP Statistical Discovery LLC, Cary, NC, USA). A significance level of 0.05 (two-tailed) was applied for all analyses.

The interactive tool for estimating the risk of recurrence in neoadjuvant-treated breast cancer patients is based on the current research results. The risk estimate follows the input data given by the user as numbers or Boolean types. The process is totally anonymous, and no sensitive patient data are addressed. Calculation method applies a decision tree method, and the resulting estimate represents cumulated risk in relation to the current cohort. The tool does not use any database enabling open access. Hence, no privacy statement or data protection methods are required to meet the General Data Protection Regulation (GDPR) requirements.

## Results

### Predictors of disease recurrence (DR)

Older age (≥ 56 years) and inflammatory BC at time of diagnosis predicted close to 2-fold risk of DR (Table 3a).

**Table 3:** Predictors of disease recurrence in uni- and multivariate analyses

	Classification criteria	Univariate			Multivariate		
		Hazard ratio	CI95%	*p*	Hazard ratio	CI95%	*p*
Age at diagnosis	≥ 56 versus < 56	1.7	1.0–2.8	0.04			ns.
(a) Pre-NAT (*n* = 227)							
Inflammatory disease	Yes versus no	1.8	1.1–3.1	0.02	2.1	1.2–3.7	0.007
ER	< 81.5% versus ≥ 81.5%	1.7	1.0–2.7	0.04			ns.
PR	< 30% versus ≥ 30%	2.0	1.2–3.4	0.007	2.6	1.5–4.6	0.0007
Mitotic activity	≥ 13 versus < 13		ns.		2.7	1.5–4.9	0.001
HER2-amplification	No versus yes	2.0	1.1–3.4	0.02	2.2	1.2–4.2	0.01
Apoptosis	Yes versus no	1.9	1.1–3.2	0.03	2.4	1.3–4.5	0.005
(b) Post-NAT (*n* = 168)							
Breast tumor							
Nuclear grade	3 versus 1–2	2.4	1.4–4.1	0.002	2.4	1.1–4.9	0.02
Mitotic activity	≥ 13 vs. < 13	2.3	1.3–4.0	0.002	2.3	1.0–5.2	0.04
PR	< 30% versus ≥ 30%	2.1	1.1–3.9	0.02	3.5	1.8–6.6	0.0002**
Lymph node							
Node positivity	+ versus −	3.2	1.3–8.1	0.01			ns.
Node ratio*	≥ 50% versus < 50%	2.6	1.5–4.6	0.0008			ns.
Mitotic activity	≥ 13 versus < 13	2.5	1.4–4.3	0.001	1.9	1.0–3.5	< 0.05
ER	< 81.5% versus ≥ 81.5%	2.9	1.7–5.0	0.0002			ns.
PR	< 30% versus ≥ 30%	2.5	1.2–5.2	0.01	2.8	1.5–5.5	0.002**
Apoptosis	Yes versus no	2.1	1.2–3.6	0.009			ns.

*Fraction of metastatic versus all excised axillary lymph nodes

**PR < 1% versus PR ≥ 1%

Evaluating the pre-NAT specimen, ER < 81.5%, PR < 30%, lack of HER2 amplification, and the presence of apoptotic figures were associated with up to 2-fold risk for DR (Table 3a). In multivariable analysis, inflammatory disease, lower PR expression, lack of Her2-amplification, lower mitotic activity, and the presence of apoptotic figures were statistically significant independent prognosticators.

Evaluating the post-NAT residual breast tumor of non-pCR cases (*n* = 168), high nuclear grade, mitotic activity ≥ 13/10HPF, and PR < 30% indicated more than 2-fold increased risk for DR (Table 3b). Differing from pre-NAT predictors, ER, HER2 amplification, and the presence of apoptotic figures were not statistically significant in residual disease. In multivariate analysis, high nuclear grade, higher mitotic activity, and lower PR expression were independent indicators of DR.

When analyzing post-NAT axillary specimen, significant risk of DR was associated with nodal metastasis (HR 3.2) and LN ratio ≥ 50% (HR 2.6) (Table 3b). In addition, DR was associated with lower hormone receptor status, higher mitotic activity, and the presence of apoptosis in metastatic cells. In multivariate analysis, lower PR and higher mitotic activity in metastatic cells were independent indicators of DR.

### Prognosticators of breast cancer-specific mortality (BCM)

Age was a significant prognostic factor (*p* = 0.0007) of BCM (Table 4a), and patients deceased of BC were in average 7.0 years older. Also among HER2-amplified carcinomas (*n* = 92), older age and inflammatory disease predicted BCM (HR 3.7, CI 1.2–12.0, *p* = 0.03 and HR 7.2, CI 2.0–25.8, *p* = 0.003, respectively).

**Table 4:** Predictors of breast cancer-specific mortality in uni- and multivariate analyses

	Classification criteria	Univariate			Multivariate		
		Hazard ratio	CI95%	*p*	Hazard ratio	CI95%	*p*
(a) Pre-NAT (*n* = 227)							
Age at diagnosis	≥ 56 versus < 56	2.3	1.3–4.2	0.005			ns.
Inflammatory disease	Yes versus no	2.9	1.7–4.9	< 0.0001	2.9	1.6–5.1	0.0004
Progression during NAT	Yes versus no	2.7	1.3–5.7	0.009			ns.
Histological grade	3 versus 1–2	2.0	1.1–3.5	0.02			ns.
Nuclear grade	3 versus 1–2	1.9	1.0–3.5	0.04			ns.
ER	< 81.5% versus ≥ 81.5%	1.8	1.1–3.1	0.03			ns.
PR	< 30% versus ≥ 30%	2.5	1.4–4.4	0.002	2.5	1.3–4.8	0.004
Apoptosis	Yes versus no	2.0	1.1–3.8	0.03			ns.
(b) Post-NAT (*n* = 168)							
Breast tumor							
Histological grade	3 versus 1–2	2.7	1.5–4.8	0.0008			ns.
Nuclear grade	3 versus 1–2	3.3	1.8–6.1	0.0001	2.0	1.0–4.0	0.04
LVI	Yes versus no	2.2	1.3–3.8	0.003			ns.
Mitotic activity	≥ 13 versus < 13	3.2	1.8–5.7	< 0.0001			ns.
ER	< 81.5% versus ≥ 81.5%	2.2	1.2–4.1	0.01			ns.
PR	< 30% versus ≥ 30%	2.6	1.2–5.4	0.01	3.8	1.9–7.4	0.0001**
Apoptosis	Yes versus no	2.1	1.1–3.9	0.02			ns.
Tumor necrosis	Yes versus no	2.7	1.4–5.2	0.004			ns.
Lymph node							
Node ratio*	≥ 50% versus < 50%	2.7	1.4–5.0	0.002			ns.
Mitotic activity	≥ 13 versus < 13	4.0	2.2–7.4	< 0.0001	2.6	1.2–5.3	0.01
ER	< 81.5% versus ≥ 81.5%	3.3	1.8–6.1	< 0.0001			ns.
PR	< 30% versus ≥ 30%	3.1	1.3–7.4	0.01	3.1	1.5–6.6	0.003**
Ki-67	≥ 30% versus < 30%	3.0	1.5–5.8	0.001			ns.
Apoptosis	Yes versus no	2.9	1.6–5.3	0.0004			ns.

*Fraction of metastatic versus all excised axillary lymph nodes

**PR < 1% versus PR ≥ 1%

**Fig. 1:**
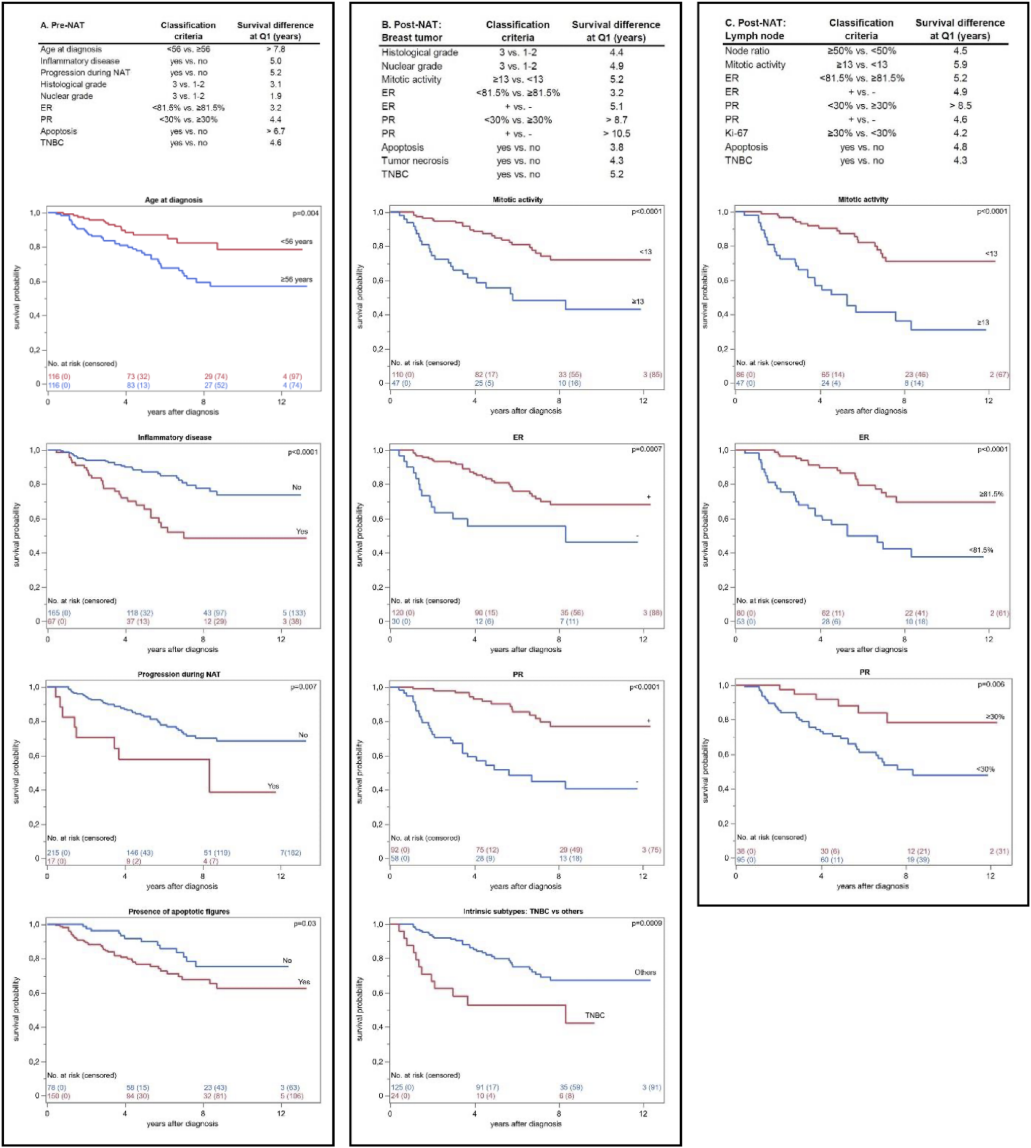
Summary of statistically significant (*p* < 0.05) Kaplan–Meier analyses representing estimations for survival differences (years) at 75% quartile (Q1) in pre- (**A**) and post-NAT settings (**B**, **C**). Features associated with > 5-year Q1 survival difference are highlighted with survival curves

Evaluating the pre-NAT specimen, high histological and nuclear grade, the presence of apoptotic figures, and lower hormone receptor expression predicted up to 2.5-fold risk of BCM (CI 1.4–4.4, *p* = 0.002 for PR < 30%). In Kaplan–Meier analyses, the most prominent survival differences were observed for age and the presence of apoptotic figures (Fig. 1a). Demonstrated with quartile estimations, the majority, 75% of patients (Q1) lacking apoptotic cancer cells had a 6.7-year shorter expected survival time as compared to cases with apoptotic figures. In multivariate analysis (Table 4a), inflammatory disease and lower PR predicted BCM, while the prognostic impact of apoptotic figures sparsely failed to show statistical significance (*p* = 0.05).

Evaluating the post-NAT residual breast tumor, the highest HRs were associated with proliferation ≥ 13 mitoses/10HPF and high nuclear grade indicating over threefold increased risk of BCM (Table 4b). In quartile estimations of Kaplan–Meier analyses (Fig. 1b), the highest survival difference at Q1 was associated with PR-negativity predicting over 10.5-year shorter survival time. In multivariate analysis, independent prognostic impact was associated with high nuclear grade and low PR.

Evaluating post-NAT axillary metastases, LN ratio (*p* = 0.002) predicted BCM, while lymph node status (+ vs. -−) sparsely failed to show prognostic significance (*p* = 0.05) (Table 4b). Higher proliferation and lower hormone receptor expression in metastatic cells were associated with up to 4.0-fold mortality risk. In Kaplan–Meier analyses (Fig. 1c), a 8.5-year survival difference at Q1 was observed for lower vs. higher PR expression. In multivariate analysis, lack of PR expression and higher mitotic activity in metastatic cells showed independent prognostic impact.

### Impact of histopathological feature changes following NAT

Histopathological feature (Table 2) changes from pre- to post-NAT are summarized in Supplementary Table 2. The most prominent alterations were detected for Ki-67 labeling index, which decreased in 39.7% of cases. A decrease of PR expression was observed in 24.9% of cases. Changes observed in mitotic activity were statistically significantly associated with prognosis. Tumors with increasing mitotic activity (from pre-NAT < 13 to post-NAT ≥ 13/10HPF) showed increased risk for recurrence (HR 3.3, CI 1.6–7.0, *p* = 0.001) and mortality (HR 3.8, CI 1.7–8.5, *p* = 0.001) indicating a 3.2-year shorter survival in quartile estimations (Q1) of Kaplan–Meier analyses (*p* = 0.01). The risk for unfavorable disease outcome was pronounced if high (≥ 13/10HPF) mitotic activity remained unchanged from pre-NAT breast tumor to post-NAT lymph node (HR 8.5, CI 1.1–65.5, *p* = 0.04 for disease recurrence and HR 4.8, CI 1.1–21.6, *p* = 0.04 for mortality).

## Discussion

Despite the era of precision medicine, there are limited data on the prognostic impacts of histopathological features and their alterations following NAT [14]. Achieving pCR is an established favorable prognostic sign but—as a reflection of the heterogeneous nature of breast cancer—the fraction of pCR varies greatly in patient subgroups ranging from 50–80% in Her2-amplified to 5–20% in luminal BC [28]. Maximizing the therapeutic benefit of NAT calls for intensified validation of the prognostic impact but even clinically applied prognostic features suffer from lack of reliable threshold values, especially in distinct breast cancer subtypes [29].

**Fig. 2:**
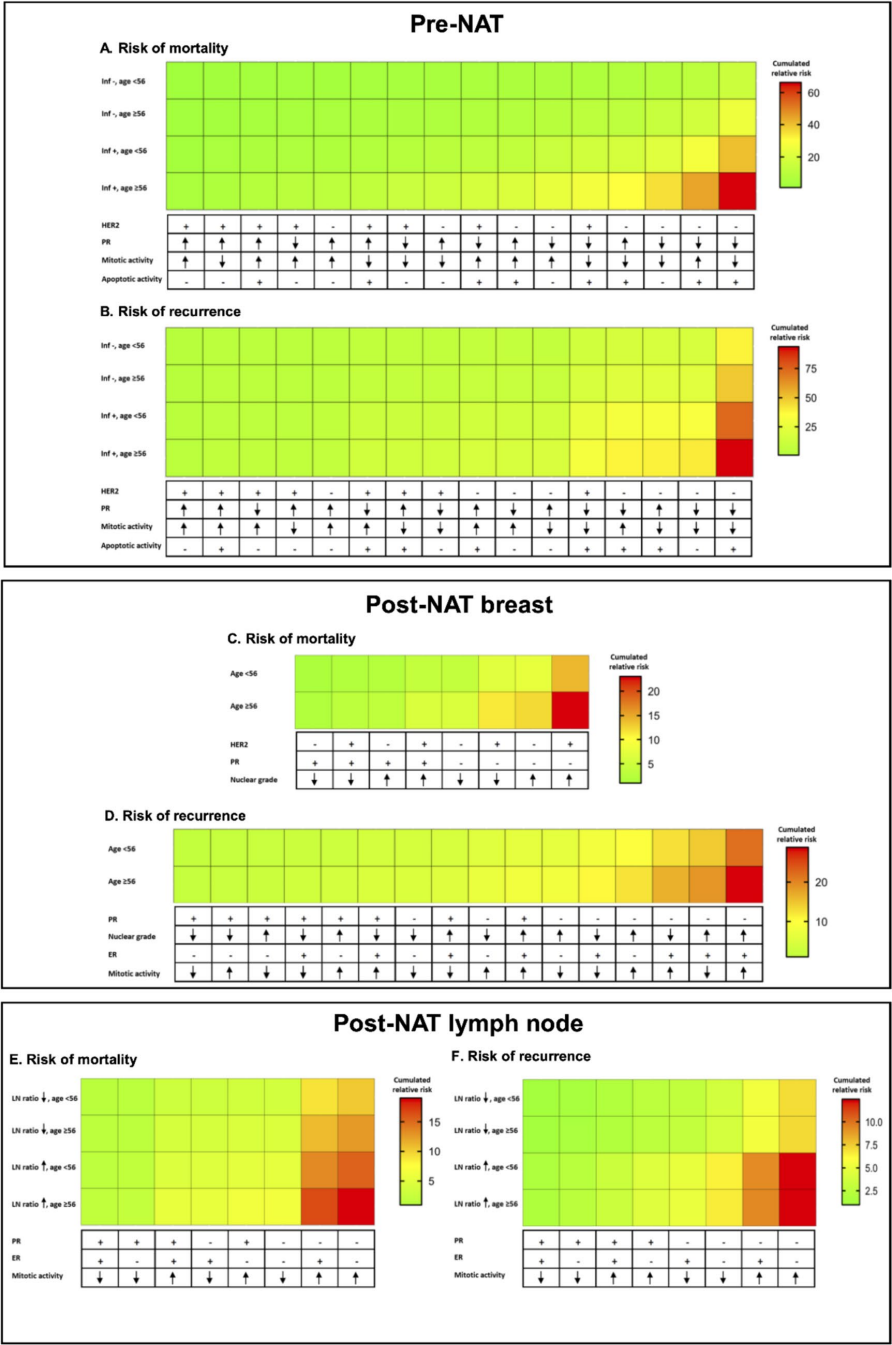
Heat maps demonstrate the relative increase of risks for mortality (**A**, **C**, **E**) and recurrence (**B**, **D**, **F**) associated with each combination of prognosticators in pre- and post-NAT settings

To illustrate the relative risks for breast cancer mortality and recurrence, the identified clinical and histopathological prognostic features were compiled into heat maps (Fig. 2). The recurrence risks derived from pre-NAT prognosticators were exemplified as an interactive tool (bcnatreccalc.utu.fi) to demonstrate the spectrum of outcomes in the different patient groups.

Age at diagnosis predicted disease outcome. Also previous literature reports on survival differences between younger vs. older NA-treated BC patients [30, 31]. However, the prognostic impact of age is complex and dependent on tumor subtype [31]. Particularly, younger women with aggressive tumor features are at a higher risk of advanced disease, recurrence, and mortality [32], while the same features predict favorable response to NAT [33]. Similarly in our results, the subgroup of breast cancer patients younger than 56 years at diagnosis had a high probability of achieving positive NAT-response and favorable outcome of disease.

NAT is the standard treatment of inflammatory BC regardless of lymph node status. Inflammatory BC is characterized by a particularly aggressive course of disease with high propensity for disease recurrence and mortality [34] and, as an initially unresectable disease, the outcome is critically dependent on NAT-response. Comparable to earlier reports [35], we observed similar pCR rates for patients with (23.7%) and without (25.8%) inflammatory disease. In line with previous literature [36], inflammatory disease in Her2-amplified tumors predicted 7.5-fold increased mortality risk (*p* = 0.002, CI 2.1–26.9). As a clinical diagnosis, the prognostic impact of inflammatory BC is limited by the lack of detailed objective diagnostic criteria [37, 38].

Higher mitotic activity is associated with pCR. This reflects the anti-mitotic major mechanism of action of taxanes: inhibition of metaphase progression leading to defective spindle formation, cell cycle arrest, and apoptosis [39]. In line with previous literature [40, 41], high mitotic activity both in the residual breast tumor and in lymph node metastasis predicted sinister course of disease, reaching 8.5-fold risk of BC death. A more favorable outcome was observed for tumors with NAT leading to decreased mitotic activity. The threshold at 13 mitoses/10HPF has also previously been recognized optimal for prognostic associations [41]. Despite the observed prognostic significance, mitotic score is not routinely re-evaluated in post-NAT specimen. The Ki-67 proliferation index correlates with, but is not equivalent to mitotic score [42]. In previous literature, Ki-67-IHC has been observed as a predictive feature for pCR [40], whereas high Ki-67 labeling index in residual lymph node metastases may predispose to chemotherapy resistance [43]. However, in our material, Ki-67 labeling index was not an independent prognosticator.

The presence of apoptotic figures had a strong and independent prognostic impact for disease outcome. The dysfunction of apoptosis regulators comprises a crucial oncogenic mechanism [44]. Apoptosis induction comprises a successful non-surgical treatment, utilized e.g., in taxanes, a key group of NA-drugs [39, 45]. Still, apoptoses are not routinely applied in prognostic evaluations. In our results, even a single morphologically observed apoptotic body predicted a doubled risk for disease recurrence and mortality. Apoptotic cell death is considered a tumor suppressor mechanism. However, there is evidence on the pro-oncogenic and drug resistance effects of apoptotic cells. In malignancy, apoptosis may paradoxically promote tumor growth and metastasis, potentially through ER-dependency or interactions with tumor microenvironment [46, 47]. An intriguing question is whether apoptosis-induced treatment resistance develops due to existing molecular mechanisms or whether aggressive cell clones develop new changes during treatment.

The prognostic value of PR outweighed the impact of ER. Lower PR immunoexpression predicted more than 3-fold increased risk of BC death. In HER2-positive BC, PR status has been described a strong predictor of NAT-response [48]. Furthermore, ER+/PR− BCs have been associated with beneficial NA-response, yet an unfavorable course of disease [49]. Still, the prognostic role of PR remains less defined than ER, and in clinical practice, PR status does not routinely drive NAT or adjuvant treatment decisions [50, 51]. Previously, PR has been associated with epithelial cell proliferation and stem cell regulation both in normal breast and breast cancer [52]. PR+ BCs might be ER-dependent and regulated through a functional ERα signaling pathway [46, 53]. This far, attempts to develop PR-targeted therapies have achieved limited progress.

HER2 amplification was associated with longer disease-free survival. Previously, pCR achieved after anti-Her2-treatment has been associated with more favorable course of disease [30, 33, 54]. Conversely, residual disease, particularly nodal metastasis following Her2-targeted NAT comprises a therapeutic challenge [13, 30]. Incomplete treatment outcome has been explained by intratumoral heterogeneity [7], while part of previous research suggests the role of reduced HER2 expression following NAT [55].

Lymph node status is the cornerstone of breast cancer prognostication [19]. In our results, the prognostic value of LN ratio outweighed metastatic status. Previous literature suggests that LN ratio may add valuable prognostic information for NA-treated BC patients [22]. In a meta-analysis of 4864 patients, high LN ratio was significantly associated with short overall survival and breast cancer-specific mortality [56]. In our material, patients with LN ratio > 50% were associated with at least 2.6-fold risk for recurrence and mortality. In addition, LN ratio had the potential to identify sinister outcome among patients with generally favorable course of disease. Based on our observations, LN ratio provides a method to intensify prognostic evaluations among patients with node positive disease.

In the present study, the generalizability of the findings may be limited by the inclusionary decision to investigate a consecutive retrospective cohort of NA-treated patients. During the study period, the indications for NAT were uniform but the clinical utility of the results might be hampered due to the real-life nature of the cohort. Lack of specific symptoms or definitive diagnostic test modalities leads to challenges in detecting and timing BC recurrence. Due to the constraints of the sample size, not all potential differences may have been captured, and the extensive testing may have introduced some false positive associations. The risk estimates of the interactive tool are mathematically calculated to exemplify relative risks representing the present data, and—prior to further validation in external cohorts—should be interpreted with caution.

In conclusion, comprehensive evaluation of conventional patient characteristics and biomarkers provides potential to optimize personalized NAT strategies. The results highlight the independent prognostic value of previously overlooked features, e.g., apoptotic activity, PR and LN ratio. The prognostic value of alterations in biomarker expressions, particularly increased mitotic activity following NAT, emphasizes the importance of re-assessment of residual breast and metastatic specimen. An interactive risk assessment tool demonstrates the continuum of different disease outcomes, and may provide additional evidence for follow-up and adjuvant interventions.

## Supplementary Information

Below is the link to the electronic supplementary material.

Supplementary file1 (PDF 381 KB)

## Data Availability

The datasets used in this study are available from the corresponding author upon a reasonable request.
