# Supplementary material for: Personalized multifactorial risk assessment in neoadjuvant-treated breast carcinoma
Source: Breast Cancer Res Treat. 2024 Dec 30;210(2):463–75. doi: 10.1007/s10549-024-07584-4 (PMC11930868; doi:10.1007/s10549-024-07584-4)
Supplement: Supplementary file 1 — Supplementary file1 (PDF 381 KB) [file 10549_2024_7584_MOESM1_ESM.pdf]

Supplementary material

Personalized multifactorial risk assessment in neoadjuvant-treated breast carcinoma

Korpinen K[1], Autere TA[1], Tuominen J[2], Löyttyniemi E[3], Eigeliene N[4], Talvinen K[1], Kronqvist P[1,2].

Institute of Biomedicine[1], University of Turku, and Department of Pathology[2], Turku University Hospital, Turku, Finland. Department of Biostatistics[3], University of Turku, Turku, Finland. Department of Oncology[4], Vaasa Central Hospital, Vaasa, Finland.

Corresponding author:

Katarina Korpinen, MD; Institute of Biomedicine, University of Turku, Kiinamyllynkatu 10/MedD5A, 20500 Turku, Finland.

Email: kkhkor@utu.fi.

ORCID: 0000-0003-4833-2613

Supplementary Table 1 Summary of oncological and surgical procedures (n=257)

Neoadjuvant (%)	
Cytostatic drug	
Taxane	67.2
Taxane and antracycline	26.5
Antracycline	2.3
Other cytostatic	0.8
Anti-HER2	39.3
Platinum-containing	9.3
Capesitabine	4.7
Hormonal	1.9
**Surgery (%)**	
Mastectomy	95.4
Breast conserving	4.6
Axillary evacuation	90.1
Sentinel lymph node biopsy	8.4
**Adjuvant (%)**	
Radiation therapy	88.9
Hormonal	67.7

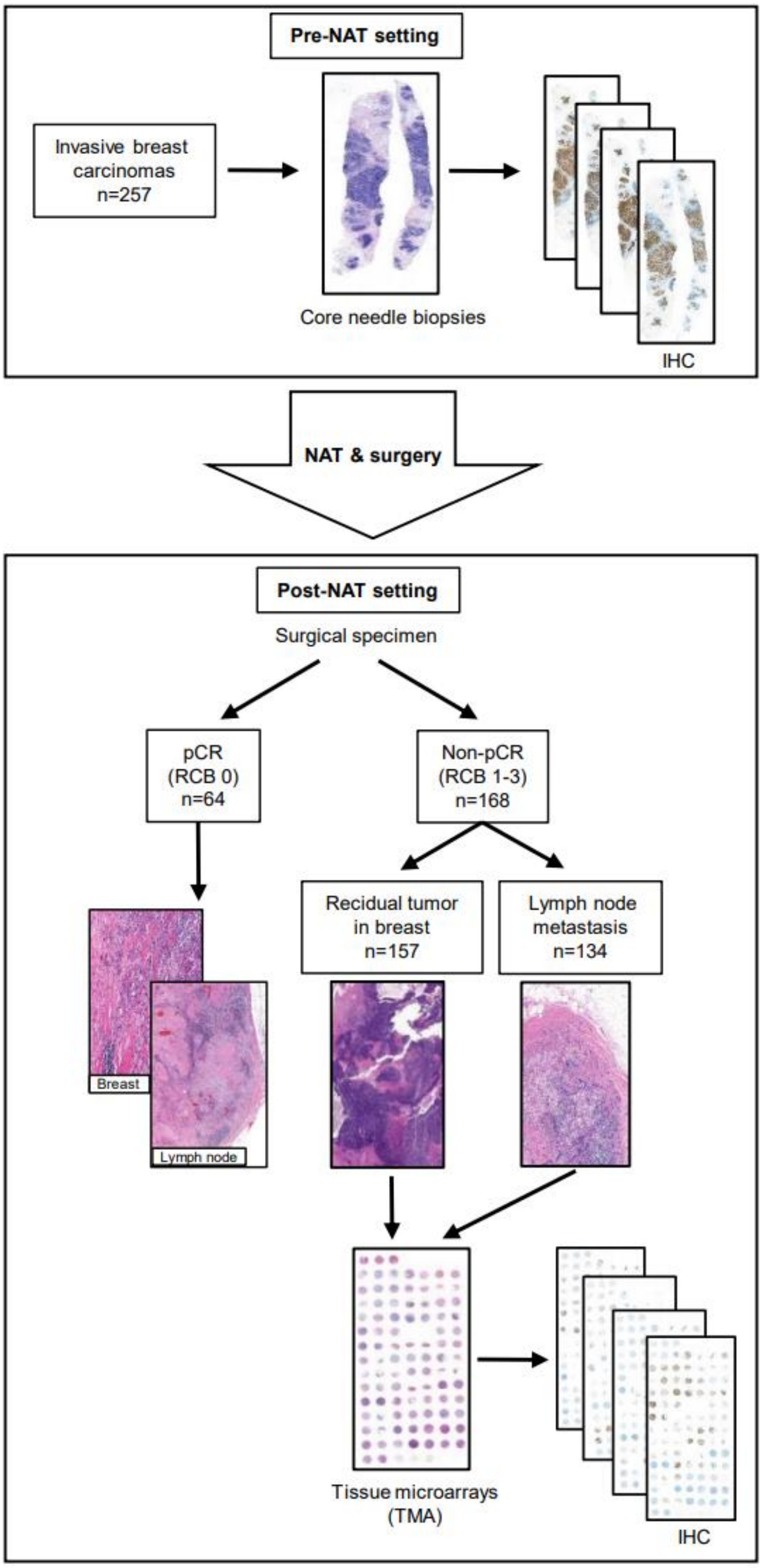

Supplementary Figure 1 Schematic representation of patient and tissue materials with examples of histological specimen and immunohistochemical (IHC) stainings in the pre- and post-NAT settings.

Korpinen et al. Personalized multifactorial risk assessment in neoadjuvant-treated breast carcinoma.

Breast Cancer Res Treat 2024.

Supplementary Table 2 Summary of alterations in histopathological features following NAT (n=181)

Tumor characteristics	Alteration pre-NAT	→ post-NAT	Breast pre-NAT vs. post-NAT % (n)	Breast pre-NAT vs. metastasis % (n)
Nuclear gradus	high*	low*	18.2 (33)	
	low*	high*	7.7 (14)	
Gradus	high*	low*	18.2 (33)	
	low*	high*	6.1 (11)	
Mitotic activity	≥13	<13	11.6 (21)	10.5 (16)
	<13	≥13	12.2 (22)	17.8 (27)
ER	+	-	4.6 (8)	8.5 (13)
	-	+	2.9 (5)	3.9 (6)
	≥81.5%	<81.5%	13.2 (23)	12.4 (19)
	<81.5%	≥81.5%	7.5 (13)	9.2 (14)
PR	+	-	13.9 (24)	19.6 (30)
	-	+	3.5 (6)	2.0 (3)
	≥30%	<30%	24.9 (43)	31.4 (48)
	<30%	≥30%	1.7 (3)	1.3 (2)
HER2-amplification	+	-	10.5 (18)	7.9 (12)
	-	+	0	0.7 (1)
Apoptosis	+	-	5.0 (9)	8.6 (13)
	-	+	34.3 (62)	22.4 (34)
Ki-67	≥30%	<30%	39.7 (69)	33.3 (51)
	<30%	≥30%	1.7 (3)	1.3 (2)
Intrinsic classification				
	LumA	LumB Her2-	0.6 (1)	1.3 (2)
	LumB Her2-	LumA	36.0 (62)	35.1 (53)
		Her2+		0.7 (1)
		TNBC	4.7 (8)	6.0 (9)
	LumB Her2+	LumA	5.8 (10)	3.3 (5)
		LumB Her2-	2.9 (5)	1.3 (2)
		Her2+	0.6 (1)	1.3 (2)
		TNBC	0.6 (1)	1.3 (2)
	Her2+	LumB Her2-		0.7 (1)
		LumB Her2+	1.2 (2)	1.3 (2)
		TNBC	1.2 (2)	1.3 (2)
	TNBC	LumA	0.6 (1)	
		LumB Her2-	0.6 (1)	1.3 (2)

* low grades 1 and 2, high grade 3
